# Opinion: exploring alternative pathways to neuroprotection—nicotine and carbon monoxide as antioxidative factors in neurodegeneration and delirium

**DOI:** 10.3389/fneur.2025.1556456

**Published:** 2025-04-09

**Authors:** Marcos Delgado, Reto A. Schuepbach, Jan Bartussek

**Affiliations:** ^1^Institute of Intensive Care Medicine, University Hospital Zurich and University of Zurich, Zurich, Switzerland; ^2^Department for Quantitative Biomedicine, University of Zurich, Zurich, Switzerland

**Keywords:** delirium, oxidative stress, nicotine, carbon monoxide, nicotinic receptors, neurodegenerative diseases

## 1 Introduction

### 1.1 Oxidative mechanisms underlying neurodegeneration

Oxidative stress arises from the excessive production of toxic free radicals, known as reactive oxygen species (ROS), regularly generated as byproducts of mitochondrial ATP production. When the balance between ROS generation and antioxidant defenses, such as glutathione, is disrupted, ROS will accumulate, leading to cellular damage (1). Oxidative stress leads to lipid peroxidation of neuronal membranes, protein damage, and DNA/RNA oxidation, triggering cellular apoptosis and promoting the release of inflammatory cytokines, which play a key role in the pathogenesis of neurocognitive disorders (2). These free radicals cause neuronal protein and genetic dysfunctions, leading to protein aggregation (such as Lewy bodies, amyloid plaques, or neurofibrillary tangles) and iron accumulation in the brain—common pathological features of neurocognitive disorders like Parkinson's disease (PD), Alzheimer's disease (AD), amyotrophic lateral sclerosis (ALS), and prion diseases (2).

### 1.2 The neuroprotective effect of smoking

Smoking is a major public health concern due to its association with high morbidity and mortality. Numerous studies have demonstrated its strong correlation with cardiovascular, respiratory, and cancer-related diseases, ultimately increasing mortality rates and healthcare costs (3). However, this contrasts with extensive epidemiological research suggesting an inverse correlation between smoking and the incidence of neurodegenerative diseases like PD and AD, while not negating the higher mortality rate among smokers (4, 5). This observation suggests that certain compounds in tobacco smoke may offer potential therapeutic benefits.

## 2 Discussion

### 2.1 Nicotine as neuroprotective factor

Nicotine is the most abundant and extensively studied components of tobacco. Numerous studies have shown that neurodegenerative diseases are associated with a reduction in nicotinic acetylcholine receptors (nAChRs) (6).

#### 2.1.1 Nicotinic acetylcholine receptor upregulation

Nicotine's interaction with α7 and α4β2-nAChRs, leading to their upregulation and an increase in binding sites, may underlie its neuroprotective effects in patients with PD and AD. This mechanism could contribute to both symptomatic relief and improved cognitive function (7). Radiological and postmortem studies have shown a reduction in nAChR density in individuals with these neurodegenerative diseases (6, 8). These findings support the hypothesis that enhancing nAChR expression, may be one mechanism by which nicotine provides neuroprotection, potentially reducing or delaying disease onset.

#### 2.1.2 The antioxidant effect of nicotine

**Figure 1:**
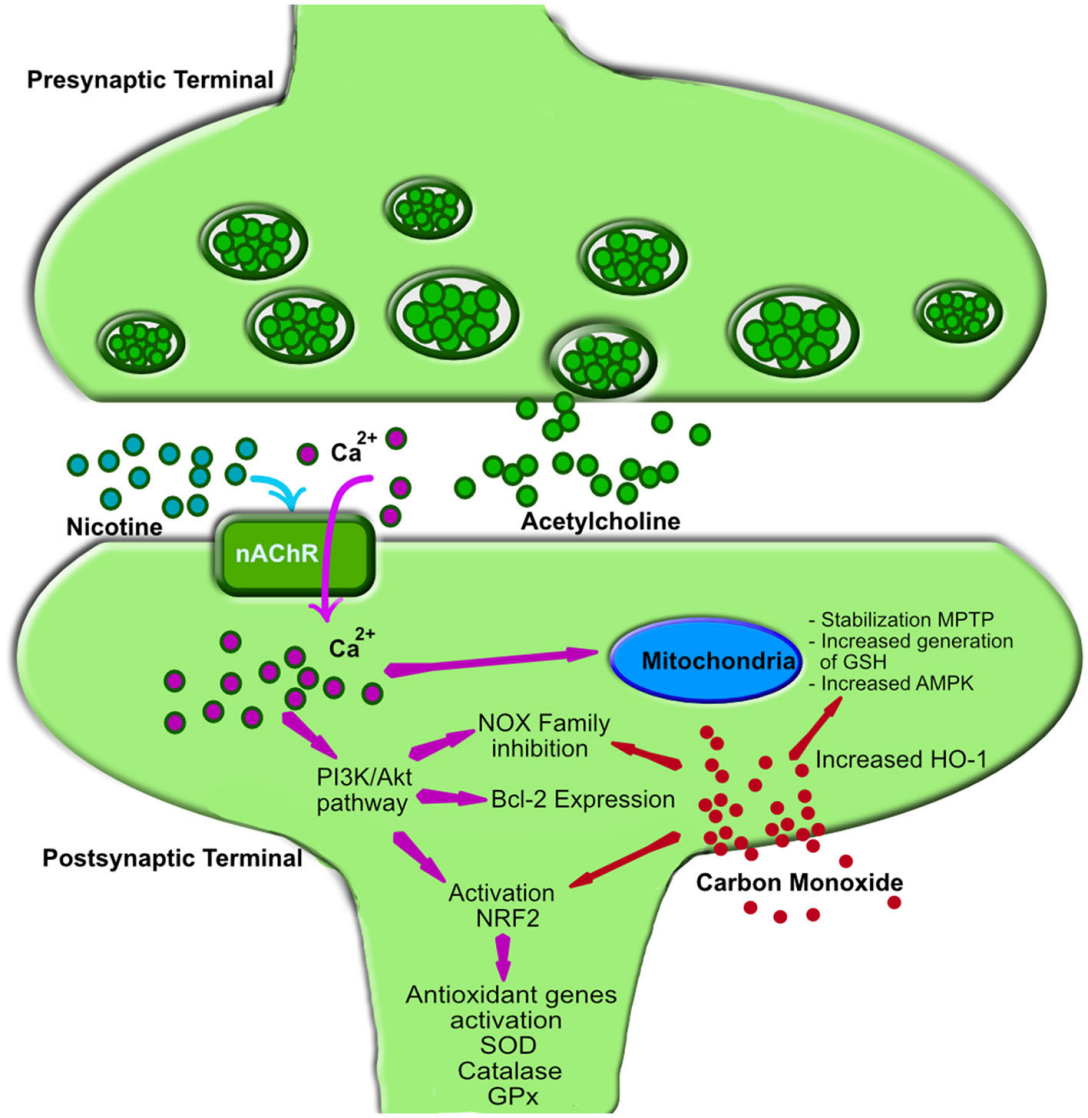
Mechanism of action of nicotine and carbon monoxide in oxidative stress reduction. Nicotine interacts with nicotinic acetylcholine receptors (nAChRs), triggering calcium (Ca^2+^) influx into the postsynaptic membrane of neurons (32). The increased intracellular Ca^2+^ activates the phosphoinositide 3-kinase (PI3K) and protein kinase B (PKB or Akt) signaling pathways (32), leading to the following effects: (1) Activation of Nuclear Factor Erythroid 2-Related Factor 2 (NRF2): This upregulates antioxidant genes such as superoxide dismutase (SOD), catalase, and glutathione peroxidase (GPx), reducing free radical accumulation (33). (2) Upregulation of B-cell lymphoma 2 (Bcl-2): Bcl-2, a key anti-apoptotic protein, protects neurons from oxidative damage by preventing mitochondrial dysfunction and inhibiting cytochrome C release (34). (3) Inhibition of NADPH oxidase (NOX) enzymes: This decreases reactive oxygen species (ROS) production, reducing oxidative stress (35). Additionally, the Ca^2+^ influx is the starting point for modulation on mitochondrial energy metabolism through: (4) Activation of AMP-activated protein kinase (AMPK): This enhances ATP production efficiency and minimizes excessive ROS generation by optimizing electron transport chain function (32). (5) Regulation of mitochondrial membrane potential, through Bcl-2 and AMPK activity: Nicotine prevents the opening of the mitochondrial permeability transition pore (MPTP), thereby mitigating uncontrolled ROS release (36). (6) NRF2-dependent mitochondrial glutathione (GSH) synthesis, through NRF2 Activation: This enhances mitochondrial antioxidant capacity and protects against oxidative damage (37). Carbon monoxide (CO), a lipophilic molecule, readily enters cells and triggers several intracellular responses, primarily leading to the upregulation of heme oxygenase 1 (HO-1) (38). (1) Similar to nicotine, CO stabilizes NRF2 (39) and activates AMPK (40), which in turn promotes HO-1 expression. (2) Additionally, CO inhibits the NOX family of enzymes, thereby reducing ROS formation. (3) The increased HO-1 production exerts antioxidant effects by degrading free heme, lowering free iron levels, and enhancing neurotrophins activity (41). nAChR, nicotinic acetylcholine receptor; PI3K, phosphoinositide 3-kinase; Akt, protein kinase B (PKB); NRF2, Nuclear Factor Erythroid 2-Related Factor 2; SOD, superoxide dismutase; GPx, glutathione peroxidase; Bcl-2, B-cell lymphoma 2; NOX, NADPH oxidase enzymes family; AMPK, AMP-activated protein kinase; MPTP, mitochondrial permeability transition pore; ROS, reactive oxygen species; GSH, mitochondrial glutathione; CO, Carbon monoxide; HO-1, heme oxygenase 1.

A substantial body of research has investigated the antioxidant properties of nicotine. Most of these studies are based on preclinical trials at the cellular, molecular, or animal level. However, also clinical, epidemiological, and postmortem studies in humans highlight the significant therapeutic potential of nicotine. A mechanistic biochemical representation of the major nicotine-related pathways that are thought to be involved in reducing oxidative stress is depicted in Figure 1.

In 1999, Linert et al. (9) investigated the potential antioxidant actions of nicotine in both *in vitro* and *in vivo* models. They analyzed the effect of iron in the substantia nigra pars compacta when combined with hydrogen peroxide (H_2_O_2_) to induce the Fenton reaction, a process that amplifies oxidative stress and is considered a key mechanism underlying Parkinson's disease. Their results suggest that nicotine can bind iron and potentially reduce its redox activity; however, direct evidence for its inhibition of the Fenton reaction remains limited. Nonetheless, this interaction could represent one pathway through which nicotine exerts antioxidative and, consequently, neuroprotective effects.

A study conducted by Guan et al. in (10) investigated the effects of varying concentrations of nicotine, along with oxidative and antioxidative substances, on the viability of a rat pheochromocytoma cell line. Cell survival was assessed using the MTT assay (3-(4,5-methylthiazol-2-yl)-2,5-diphenyltetrazolium bromide assay), a method that evaluates cellular metabolic activity based on mitochondrial function. A decrease in MTT assay readings was interpreted as indicative of cell destruction through oxidative stress mechanisms. The results demonstrated that cell viability remained stable when exposed to low concentrations of nicotine. In contrast, higher nicotine doses resulted in a reduction in cell survival. But, the co-administration of antioxidants with high nicotine doses mitigated this effect, preserving cell viability at levels comparable to control samples. Similarly, the addition of oxidative agents to the culture, combined with low-dose nicotine, maintained cell viability comparable to that of the control group. The study also explored the impact of amyloid beta peptide (Aβ25-35), a neurotoxic molecule implicated in Alzheimer's disease due to its oxidative properties. Exposure to Aβ25-35 significantly reduced cell viability; however, the administration of low-dose nicotine restored cell survival to normal levels. In contrast, increasing the nicotine dose led to a marked reduction in cell viability. These findings underscore the dual role of nicotine, with low concentrations exerting a protective effect under conditions of oxidative stress, while higher doses are cytotoxic.

Dong and collaborators (11) conducted an interesting study evaluating the effect of low-dose nicotine on hippocampal neurons under the hypothesis of a protective effect against Alzheimer's disease. In their research, they demonstrated that nicotine suppresses neuronal damage induced by H_2_O_2_ in hippocampal cells, reducing the generation of ROS through the activation of α7-nAchR and upregulation of Erk 1 and 2 (extracellular signal-Regulated Kinases 1 and 2), essential in promoting cell survival and proliferation and prevention of apoptosis.

Another recent trial conducted by Boiangiu et al. (12) using rat models of AD showed that cotinine and 6-hydroxy-L-nicotine, both nicotinic derivatives, improve memory and reduce oxidative stress by modulating nicotinic acetylcholine receptors (nAChRs). These substances enhance spatial and recognition memory, decrease acetylcholinesterase activity, and restore antioxidant defenses in the hippocampus, suggesting their potential as treatments for cognitive deterioration.

For ethical reasons, clinical trials assessing nicotine at different doses have not been possible. However, the reviews by Fratiglioni (4), Allam (5) or Picciotto (13) provide a comprehensive summary of several epidemiological, imaging, and postmortem studies investigating the mechanisms of nicotine's neuroprotection. Some of these trials are summarized in Table 1. Gorell et al. (14) reported a clear negative association between smoking and PD, with a dose-dependent neuroprotective effect that diminishes over time after smoking cessation. While the neuroprotective effect of nicotine in PD is well established, its role in AD remains controversial. Although preclinical studies suggest a protective effect in AD, this is challenged by the cerebrovascular ischemia associated with smoking (4) and other studies suggesting neuroinflammatory, oxidative stress mechanism and mitochondrial dysfunction in the pathophysiology of AD (8). In contrast, postmortem studies comparing elderly smokers and non-smokers have shown a significant reduction in amyloid-beta plaques in smokers (15), suggesting a potential protective effect against AD. This finding may also be linked to the upregulation of nAChRs in the brains of smokers with AD (16).

**Table 1:** Overview of selected studies.

**Trials**	**Method**	**Findings**	**References**
**Pre-clinical**			
*Linert W et al. Biochim Biophys Acta. 1999* Klicken oder tippen Sie hier, um Text einzugeben.	Sprague-Dawley rats treated with nicotine to assess the Fenton reaction in the substantia nigra pars compacta.	Nicotine is suggested to form a nicotine-iron complex, preventing the Fenton reaction and reducing ROS generation.	(9)
*Guan ZZ et al. Neurochem Int. 2003* Klicken oder tippen Sie hier, um Text einzugeben.	PC-12 pheochromocytoma cell line from rats exposed to oxidant/antioxidant substances, with cell viability assessed via MTT assay.	Low nicotine concentrations reduce lipid peroxidation and prevent the loss of nicotinic receptor binding sites caused by amyloid β. High nicotine concentrations decrease cell viability and increase lipid peroxidation.	(10)
*Dong Y et al. Front Mol Neurosci. 2020* Klicken oder tippen Sie hier, um Text einzugeben.	HT-22 cells pretreated with a low dose of nicotine and challenged with selective inhibitors.	Activation of α7-nAChRs/Erk1/2 signaling pathways by nicotine reduces ROS generation, suppressing oxidative cell injury.	(11)
*Boiangiu RS et al. Antioxidants. 2020* Klicken oder tippen Sie hier, um Text einzugeben.	Administration of cotinine and 6-hydroxy-L-nicotine in Aβ 25-35-treated rat model of AD.	Reduction in acetylcholinesterase activity and modulation of nAChRs in hippocampal tissue improve cognitive deficits.	(12)
*Rose KN et al. bioRxiv*. 2024 Klicken oder tippen Sie hier, um Text einzugeben.	Rodent models of PD with α-synuclein (Lewy body) accumulation exposed to a low dose of carbon monoxide.	Activation of the heme oxygenase-1 cascade promotes α-synuclein degradation and reduces dopamine cell loss.	(19)
**Clinical**			
*Gorell J et al. Neurology. 1999* Klicken oder tippen Sie hier, um Text einzugeben.	Retrospective case-control assessment.	Dose-dependent effect of smoking on reducing PD incidence.	(14)
*Mousavi M et al. Neuroscience. 2003* Klicken oder tippen Sie hier, um Text einzugeben.	Brain autopsy analysis using Western blot and RT-PCR to assess nAChR mRNA levels in smokers and non-smokers, with and without AD.	α4 and α7 nAChR subunits are increased in the temporal cortex of smokers, including those with AD.	(16)
*Alves G et al. Mov Disord. 2004* Klicken oder tippen Sie hier, um Text einzugeben.	Prospective study of disease progression and mortality in smoking and non-smoking patients with PD.	No significant differences in progression of parkinsonism, cognitive impairment, and mood in smoking and non-smoking patients with PD.	(17)
*Court JA et al. Neuropathol Appl Neurobiol. 2005* Klicken oder tippen Sie hier, um Text einzugeben.	Neuropathological postmortem brain assessment comparing smokers and non-smokers.	Smokers showed a significantly lower density of total amyloid β plaques compared to non-smokers.	(15)
*Komninou MA et al. Front Psychiatry. 2024* Klicken oder tippen Sie hier, um Text einzugeben.	Retrospective study assessing postoperative delirium in non-smokers, active smokers, and former smokers.	Active smokers and non-smokers exhibit similar rates of postoperative delirium, while former smokers show a significantly higher incidence.	(31)

Despite these findings on nicotine in neurocognitive diseases, its role remains unclear regarding dose safety, administration frequency, intervals, and its preventive or therapeutic effects. This uncertainty is highlighted by Alves et al. (17), who found no benefit of smoking on PD progression once the disease was already established, and by Nielsen et al. (18), who demonstrated that malondialdehyde—a well-established biomarker of oxidative stress and a product of lipid peroxidation—is present at significantly higher levels in the plasma of active smokers compared to non-smokers.

### 2.2 Carbon monoxide as neuroprotective antioxidant factor

Although nicotine remains the primary focus of research on neuroprotection in neurocognitive disorders, a recent study by Rose et al. (19) provides valuable new insights of carbon monoxide (CO), another component of tobacco smoke, that open new therapeutic avenues in Parkinson's disease. Oxidative stress in PD underlies the formation and spread of α-synuclein (αSyn)-rich aggregates, known as Lewy bodies, which are thought to induce degeneration of dopaminergic neurons in the substantia nigra pars compacta, a hallmark feature of the Parkinson's disease. Rose et al. used rodent models of Parkinson's disease driven by αSyn accumulation and oxidative stress to demonstrate that CO reduces neurodegeneration and αSyn pathology by activating heme oxygenase-1 (HO-1)-mediated pathways (Figure 1). These pathways limit oxidative stress and promote the degradation of αSyn aggregates, mitigating key drivers of Parkinson's disease pathology. A particularly striking aspect of the study is the emphasis on using low doses of carbon monoxide, carefully maintained well below neurotoxic levels, to achieve these protective effects.

Consequently, low-dose carbon monoxide emerges as a potential protective factor against the destruction of dopaminergic neurons in the substantia nigra pars compacta, providing a novel therapeutic avenue for neuroprotection in Parkinson's disease.

### 2.3 The role of oxidative stress in pathogenesis of delirium

Unlike the gradual, progressive decline seen in neurodegenerative diseases, delirium is characterized by an acute onset and fluctuating course, yet both conditions may involve disruptions in neurotransmitter systems and inflammatory pathways, providing a possible common ground for investigating protective effects. Delirium is widely recognized as a multifactorial syndromic manifestation that remains challenging to categorize comprehensively. It is a frequent and life-threatening complication, particularly in Intensive Care Units (ICU), where it commonly arises in post-operative patients (20). Despite its acute and often reversible nature, delirium carries significant morbidity and mortality risks, underscoring the urgency of understanding its pathophysiological mechanisms and identifying effective preventive and therapeutic strategies (21). Growing evidence suggests that oxidative stress may underlie the molecular mechanisms responsible for neuronal dysfunction in delirium (22). In the postoperative and ICU setting, oxidative stress can be triggered by a variety of factors, including hypoxemia (23), ischemia-reperfusion injury during surgery (24), hyperoxemia induced by mechanical ventilation or extracorporeal membrane oxygenation (25), systemic inflammation (26), and the metabolic effects of critical illness (27, 28), all of which may contribute to neuronal damage and cognitive dysfunction.

#### 2.3.1 Neuroprotection for postoperative delirium?

Both nicotine and CO are toxic substances; however, at low doses, they induce effects opposite to those typically associated with their toxicity. This phenomenon, known as hormesis (29), contributes to cellular survival. Therefore, it can be postulated that nicotine and CO may act synergistically to exert a neuroprotective effect in neurodegenerative diseases. This neuroprotective effect may also be apparent in delirium, a condition that, while distinct from chronic neurodegenerative diseases like PD and AD, shares overlapping features in its impact on cognition and potential mechanisms of neuronal dysfunction (7, 30).

Recently, we published a study in which we evaluated the possible relationship between nicotine consumption and the development of delirium in postoperative ICU patients (31). In contrast to previous studies, we differentiated between former smokers and non-smokers—defined as individuals who reported never having smoked in their lifetime—and compared their incidence of postoperative delirium with that of active smokers. Surprisingly, our results demonstrated that active smokers did not exhibit a higher incidence of postoperative delirium than non-smokers, despite being in a withdrawal phase. Instead, former smokers showed a significantly higher incidence of delirium. Although the underlying mechanisms were not directly investigated in our study, we hypothesize that the desensitization and upregulation of nAChRs commonly observed in nicotine users (13) may have contributed to increased neuronal vulnerability in both active and former smokers due to receptor overexpression. However, in active smokers, the presence of nicotine during the perioperative period may have exerted a neuroprotective effect, potentially mitigating the risk of delirium observed in the former smoker group.

Both nicotine and carbon monoxide in active smokers may contribute to neurofunctional stability through their antioxidant properties, potentially offering protection against both delirium and neurodegenerative diseases. Given the relatively short half-life of carbon monoxide (4 to 6 h), its levels in active smokers were likely reduced in the postoperative period during our trial. However, it is important to note that a major period of oxidative stress likely occurs during surgery, when carbon monoxide levels might still be elevated, potentially exerting protective effects at a critical time. Moreover, nicotine and its metabolites, such as cotinine, have a much longer half-life, enabling them to remain in the bloodstream for several hours to days, potentially extending their neuroprotective effects throughout the postoperative period, including during prolonged stays in the ICU.

## 3 Conclusion

Nicotine and carbon monoxide, despite their distinct mechanisms, share antioxidative properties and exhibit neuroprotective effects at low doses. Given the links between oxidative stress, delirium, and neurodegenerative diseases, these findings suggest potential therapeutic applications for preventing and managing neurodegenerative diseases and delirium. Randomized controlled trials are warranted to evaluate their controlled use in mitigating oxidative stress and exploring their broader clinical implications.
